# Milk fat globule-epidermal growth factor 8 (MFG-E8) attenuates sepsis-induced acute kidney injury by inhibiting NF-κB signaling pathway^1^

**DOI:** 10.1590/s0102-8650201900209

**Published:** 2019-02-28

**Authors:** Yang Zhao, Qian Wang, Bin Zang

**Affiliations:** I Master, Department of Critical Care Medicine, Shengjing Hospital, China Medical University, Shenyang, China. Technical procedures, interpretation of data, statistical analysis, manuscript preparation.; II Master, Department of Emergency Medicine, 4th Affiliated Hospital, China Medical University, Shenyang, China. Acquisition and interpretation of data, statistical analysis, critical revision.; III Master, Chairman and Head, Department of Critical Care Medicine, Shengjing Hospital, China Medical University, Shenyang, China. Conception and design of the study, critical revision.

**Keywords:** Acute Kidney Injury, Apoptosis, Inflammation, NF-kappa B, Mice

## Abstract

**Purpose:**

To explore the effect of milk fat globule-epidermal growth factor 8 (MFG-E8) on sepsis-induced acute kidney injury (SAKI).

**Methods:**

Male C57BL/6 mice were randomized to control, sham, CLP, CLP+PBS, and CLP+rmMFG-E8 groups. SAKI was induced by cecal ligation and puncture (CLP). Recombinant mouse MFG-E8 (rmMFG-E8) (20 μg/kg) or PBS (vehicle) was administered intraperitoneally. Blood, urine and renal tissue were collected at 24 h after CLP. Blood samples were tested for serum kidney injury biomarker and cytokines. Urine samples were collected to detect KIM-1, and NGAL. Real-time PCR was tested for Bax and Bcl-2. TUNEL staining was used to determine renal apoptosis. Western blot was used to detect the expression of Bax, Bcl-2, and proteins in the NF-κB pathway.

**Results:**

MFG-E8 alleviated SAKI by decreasing serum Cre, BUN, urine KIM-1 and NGAL and by mitigating renal pathological changes significant (p < 0.05). IL-1β, IL-6, TNF-α were significantly inhibited by MFG-E8 (p < 0.05). Apoptosis induced by SAKI was markedly suppressed by MFG-E8. Finally, MFG-E8 attenuated the activation of the NF-𝜅B signaling pathway in SAKI.

**Conclusion:**

MFG-E8 has beneficial effects on SAKI, which may be achieved by inhibiting the NF-κB pathway.

## Introduction

 Acute kidney injury (AKI) is one of the most common and severe complications of sepsis^1^; 40-50% of patients with sepsis develop AKI with a 6 to 8 folds increase in mortality^2^. About 3-50% of inpatients experience AKI^3^. Sepsis-induced AKI not only leads to the accumulation of various metabolites but also further causes severe clinical consequences. AKI is common and is associated with many adverse perioperative outcomes in surgical patients^4^. AKI carries a two-fold increased risk of end-stage chronic kidney disease within five years^5^. Despite continuous advances in treatment, including continuous renal replacement therapy, the prognosis of sepsis-induced AKI has not been fundamentally changed, and the mortality rate remains high^6^. Sepsis-induced AKI causes a substantial financial burden^7^. Furthermore, AKI increases the durations of mechanical ventilation and hospital stays^8^. These urgently require us to understand the pathogenesis of AKI in sepsis and to carry out early treatment.

 The pathophysiology of AKI in sepsis is complicated. Most AKIs are multifaceted, and several concurrent mechanisms may be at work. These mechanisms include inflammation, microcirculation obstacles, oxidative stress, cell cycle arrest, and kidney apoptosis^9^. Increasing evidence supports a pathogenic role for apoptosis in sepsis-induced AKI. If the apoptotic cells are not cleared in time, it will lead to further inflammatory reactions, which will further lead to kidney damage. The nuclear factor NF-κB pathway is considered to be a typical signal pathway that plays an essential role in regulating the expression of pro-inflammatory genes including cytokines, chemokines, and adhesion molecules.

 MFG-E8 is a lipophilic glycoprotein on the surface of milk fat globules^10^. MFG-E8 is a vital bridge molecule in the elimination of apoptosis and can promote phagocytosis of apoptotic cells by macrophages. In inflammatory diseases, the expression of MFG-E8 is often reduced, which is related to the severity of the disease and further aggravates the condition^11^. At present, there is limited research on the role of MFG-E8 in sepsis-induced AKI. A recent study had shown that MFG-E8 could improve renal function in sepsis^12^; however, its mechanism has not yet been elucidated. In the present study, we determined the renal protective effects of MFG-E8 in mice with cecal ligation and puncture (CLP)-induced sepsis and investigated whether such renal protective effects are associated with the inhibition of inflammation and NF-κB activation. 

## Methods

###  Experimental animals and grouping 

 All animal procedures were performed according to protocols approved by the Institutional Animal Care and Use Committee of China Medical University (No. 2016PS234K). 

 Male C57BL/6 mice (8-10 weeks) were purchased from the Experimental Animal Center of China Medical University. Mice were housed in a specific pathogen-free facility at the China Medical University and were fed standard laboratory chow *ad libitum*. 

 Fifty mice were randomly allocated to five groups, with 10 mice in each of the following groups: control, sham, CLP, CLP+PBS, and CLP+rmMFG-E8. Mice were anesthetized by intraperitoneal injection of 5% chloral hydrate (6 ml/kg body weight) and subcutaneously injection of butorphanol (2.5 mg/kg body weight). CLP was used as previously described^13^. A 1-cm abdominal incision was made to expose the cecum. The cecum was ligated with a 4-0 silk suture 0.5 cm from the base of the ileocecal valve. Double puncture with a 21-gauge needle was performed to perforate the cecum. A small amount of stool was extracted from both holes, and the cecum was returned to the abdominal cavity. The abdomen was then closed in two layers and 1 milliliter of resuscitative normal saline was administered subcutaneously. The CLP+rmMFG-E8 group was treated with an intraperitoneal injection of rmMFG-E8 at a dose of 20 μg/kg in 0.1 ml PBS at the same time of the CLP procedure. The vehicle group was treated with PBS. The sham group underwent the same operative procedure except for the colon ligation and puncture. Twenty-four hours after CLP, the mice were anesthetized and blood was collected from the abdominal aorta. Blood and kidney samples were collected for various measurements.

###  Chemicals and reagents 

 TNF-α, IL-1β, and IL-6 ELISA Kits were purchased from Biolegend (San Diego, CA, USA). rmMFG-E8 was purchased from R&D Systems (Minneapolis, MN, USA). Antibodies used for western blot were as follows: anti-Bcl-2 (Proteintech, Rosemont, IL, USA), anti-Bax (Proteintech, Rosemont, IL, USA), anti-ß-actin (Proteintech, Rosemont, IL, USA), and anti-histone H3 (Proteintech, Rosemont, IL, USA). A NF-κB signal pathway kit was used to determine NF-κB pathway activation (Cell Signaling Technology, Beverly, MA, USA). Blood urea nitrogen (BUN) and serum creatinine (Cre) detection kits were purchased from the Institute of Jiancheng Bioengineering (Nanjing, China). All other reagents were of analytical grade. 

###  Kidney injury marker assay 

 Blood samples were collected for to detect blood BUN and Cre levels using commercially available kits produced by the Institute of Jiancheng Bioengineering (Nanjing, China) following the instructions of the manufacturer. The concentrations of BUN and Cre were calculated by generating a standard curve. Urine samples were collected for the detection of urinary neutrophil gelatinase-associated lipocalin (NGAL) and kidney injury molecule-1 (KIM-1) levels using commercially available kits produced by Biolegend (Boster, Wuhan, China) following the instructions of the manufacturer. 

###  Pro-inflammatory cytokines 

 Blood samples were collected for the detection of TNF-α, IL-1β and IL-6 levels using commercially available kits produced by Biolegend (San Diego, CA, USA) following the instructions of the manufacturer. The concentrations of pro-inflammatory cytokines were calculated by generating a standard curve.

###  Renal histopathology 

 Mice kidneys were fixed in 4% paraformaldehyde, dehydrated in graded ethanol, embedded in paraffin and stained with periodic acid Schiff (PAS). Random fields from each section were observed at x400 magnification. Morphological changes that indicated AKI included the loss of the brush border, the vacuolization of tubular epithelial cells, and the presence of intratubular debris. The scoring system used to grade was previously described^13^. Kidney injury was assessed by the semi-quantitative analysis of renal morphological changes. Scoring criteria were as follows: 0 points, normal control tissue; 1 point, renal tubular damage area <25%; 2 points, renal tubular damage area 25% -50%; 3 points, renal tubular damage area 50% -75%; 4 points, tubular damage area 75% -100%.

###  Western blotting 

 Total proteins and nuclear proteins in renal tissues were extracted by commercially available kits (Beyotime, China) and then denatured. Protein concentrations were determined by the BCA protein estimation kit (Beyotime, China). Equal quantities of renal protein samples were separated by SDS-PAGE and then transferred onto a 0.22-μm nitrocellulose membrane. The membranes were blocked with 5% non-fat milk in Tris-buffered saline containing 0.1% Tween-20 then incubated with anti- Bcl-2 (1:1000), anti-Bax (1:1000), anti-I𝜅B𝛼 (1:1000), anti-NF-𝜅B (1:1000), anti-p-NF-𝜅B (1:1000), anti-𝛽-actin (1:1000), and anti-Histone H3 (1:1000) antibodies, respectively, at 4°C overnight. The secondary antibody conjugated with horseradish peroxidase (1:2000) was incubated for 2 h at room temperature. A chemiluminescent peroxidase substrate (ECL; Beyotime, China) was applied according to the manufacturer’s instructions.

###  Quantitative real-time PCR 

 Total RNA was extracted from renal tissues using RNAiso Plus (TaKaRa, Dalian, China) and reverse-transcribed into complementary DNA using a PrimeScriptTM RT reagent Kit with gDNA Eraser (TaKaRa, Dalian, China). An SYBR Premix Ex Taq (TaKaRa, Dalian, China) was used to perform the PCR reaction following the instructions of the manufacturer. The primer sequences are as follows:

 β-actin Forward: CGTGAAAAGATGACCCAGATCA, Reverse: TGGTACGACCAGAGGCATACAG; Bax Forward: ATGCGTCCACCAAGAAGC, Reverse: CAGTTGAAGTTGCCATCAGC; Bcl-2 Forward: AGCCTGAGAGCAACCCAAT, Reverse: AGCGACGAGAGAAGTCATCC. The comparative CT method (2^−ΔΔCt^) was used to determine the relative quantification of target genes normalized to a reference gene (β-actin). 

###  TUNEL assay 

 The presence of apoptotic cells in the kidney was assessed using a terminal deoxynucleotide transferase dUTP nick-end labeling (TUNEL) staining kit (Roche Diagnostics, Indianapolis, IN) according to the manufacturer’s instructions. Briefly, tissue sections were dewaxed, rehydrated, and placed in 0.1 M citrate buffer (pH 6.0) then exposed to 350 W microwave irradiation for 5 min. The sections were incubated with a mixture containing terminal deoxynucleotidyl transferase and fluorescence-labeled nucleotides then examined under a fluorescence microscope. Apoptotic cells were quantified by optical microscopy (Nikon ECLIPSE TE2000-U, Japan).

###  Statistical analysis 

 Data are expressed as the means ± SE and compared by one-way analysis of variance (ANOVA) and the Student-Newman-Keuls test. Differences were considered significant at p<0.05. 

## Results

###  Effect of MFG-E8 on sepsis-induced AKI 

**Figure 1:**
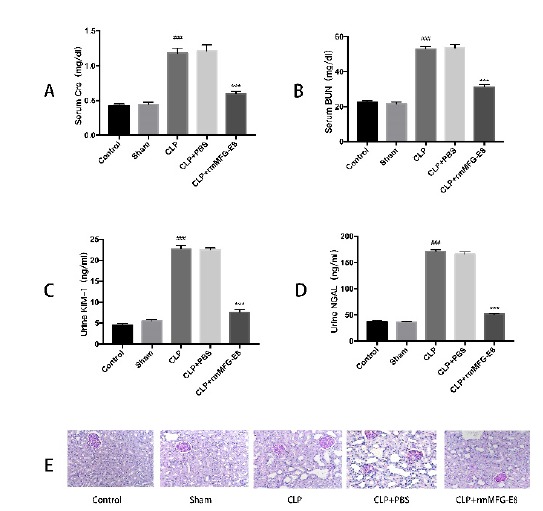
Effect of MFG-E8 on kidney injury after CLP. (**A**) Serum concentrations of creatinine (Cre) in each group 24h after CLP or sham operation. (**B**) Serum concentrations of blood urea nitrogen (BUN) in each group 24h after CLP or sham operation. (**C**) Concentrations of urine KIM-1 in each group 24h after CLP or sham operation. (**D**) Concentrations of urine NGAL in each group 24h after CLP or sham operation. (**E**) Periodic acid-Schiff (PAS) staining of kidney tissues from different groups. Magnification: ×400.***p< 0.01 compared to CLP group. ### p < 0.01 compared to Sham group.

 Serum BUN and Cre were measured to assess renal function. Serum BUN and Cre levels were significantly elevated in the CLP and CLP+PBS group; rmMFG-E8 significantly reduced both BUN and Cre levels (Fig. 1 A, B). Urine NGAL and KIM-1 can be used as early biomarkers of AKI. The results showed that urinary NGAL and KIM-1 levels were significantly elevated in the CLP and CLP + PBS groups; however, rmMFG-E8 significantly reduced urinary NGAL and KIM-1 levels (Fig. 1 C, D). To determine histopathological changes in the kidney, PAS staining was performed. As shown in Fig. 1E, renal tissues in the control group and the sham group appeared to be normal. The proximal tubule epithelial cells were vacuolated, and the epithelial cells were significantly swollen, resulting in a higher score for tubular damage in the CLP and CLP + PBS groups. Sepsis-induced epithelial disorders and interstitial edema were reduced in the rmMFG-E8 group. MFG-E8 effectively reduced pathology scores (*p*< 0.05).

###  Effect of MFG-E8 on the pro-inflammatory cytokines 

**Figure 2:**
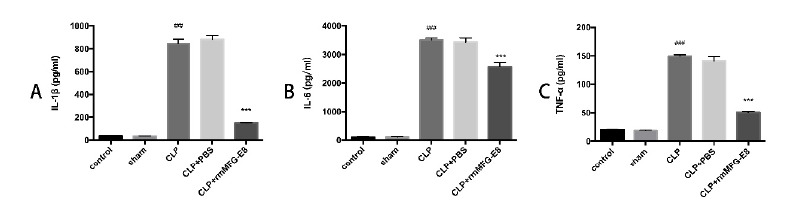
Effect of rmMFG-E8 on IL-1ß (**A**), IL-6 (**B**) and TNF-α (**C**). Quantitation of IL-1ß, IL-6, and TNF-α were performed by ELISA. Data are represented as mean±SE (n=10). *** p < 0.01 compared to CLP group. ### p < 0.01 compared to Sham group.

 Because the nature of sepsis is an excessive inflammatory response to infection, we measured serum pro-inflammatory cytokine levels including TNF-α, IL-1β and IL-6. CLP and CLP+PBS group had significantly increased levels of TNF-α, IL-1β and IL-6, whereas treatment with rmMFG-E8 inhibited the overproduction of TNF-α, IL-1β and IL-6 (TNF-𝛼: 50.81±0.7 *vs.* 149.4±1.2, *p*< 0.01; IL-1𝛽: 150.64±1.3 *vs.* 845.9±16.89, *p*< 0.01; and IL-6: 3505 ± 29.96 *vs.* 2570 ± 69.42, *p*< 0.01) (Fig. 2).

###  Effect of MFG-E8 on renal cell apoptosis 

**Figure 3:**
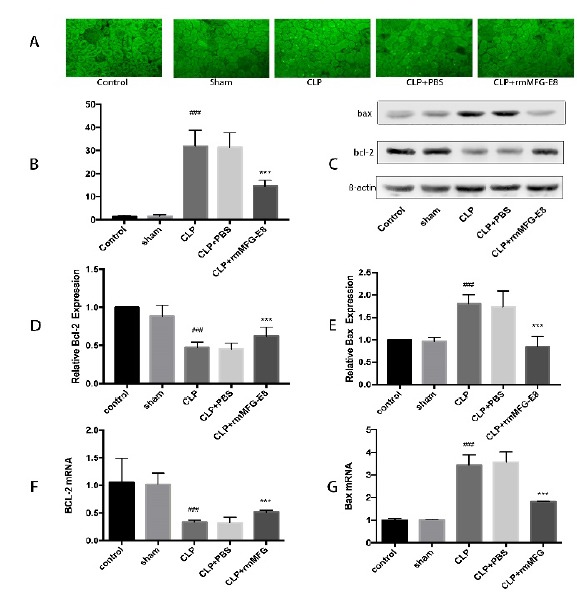
MFG-E8 inhibited the apoptosis of the renal tissue. (**A, B**) TUNEL staining and apoptotic cell counts (×400) in kidney tissues of each group. (**C, D, E**) Western blot of Bax and Bcl-2 in different groups. (**F, G**) RT-PCR of Bcl-2 and Bax in different groups. *** p < 0.01 compared to CLP group. ### p < 0.01 compared to Sham group.

 TUNEL staining was used to detect apoptosis in renal cells. The number of TUNEL-positive cells in the CLP and CLP+PBS groups were significantly increased compared with the control and sham group, and MFG-E8 treatment reduced the number of apoptotic cells (Fig. 3 A, B). To further determine the role of MFG-E8 in renal cell apoptosis, renal apoptosis-related protein and mRNA expression was determined by immunoblotting and RT-PCR, respectively. Compared with the CLP group, MFG-E8 downregulated the expression of Bax and upregulated the Bcl-2 level as shown in Fig. 3 (C-F).

###  Effect of MFG-E8 on NF-κB signal pathway in sepsis-induced AKI 

**Figure 4:**
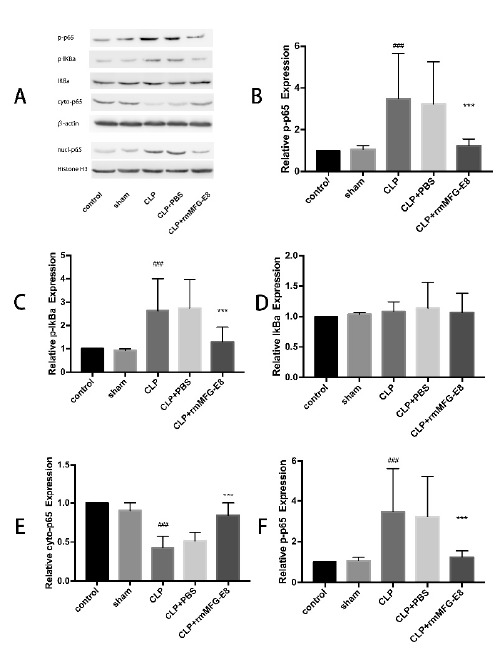
MFG-E8 inhibited the activation of NF-κB induced by CLP. The expression of related proteins of the NF-κB signaling pathway were detected by western blot. The results shown are representative of at least three independent experiments. *** p < 0.01 compared to CLP group. ### p < 0.01 compared to Sham group.

 NF-κB is an essential transcriptional regulator involved in the inflammatory response and plays an important role in sepsis. The effect of MFG-E8 on NF-κB activation was evaluated using western blotting. As shown in Figure 4, we found that the expression of p-IκB𝛼, p-p65 and NF-κB in the nucleus was significantly increased in the CLP and CLP+PBS groups. However, these AKI-induced changes were reversed by MFG-E8 treatment.

## Discussion

 Sepsis is a life-threatening disease that arises from the body’s response to systemic inflammatory response syndrome (SIRS), which causes injury to tissues and organs. It often leads to pathophysiological processes such as septic shock and multiple organ dysfunction syndrome (MODS)^14^. The kidney is one of the most vulnerable organs in sepsis. Sepsis-induced AKI occurs early, and its mortality rate is high. Therefore, it is urgent to find effective therapy to treat sepsis-induced AKI. In this study, we demonstrated that rmMFG-E8 improved renal function, inhibited pro-inflammatory factors, and achieved a protective effect on sepsis-induced AKI. 

 A series of pathological processes are involved in the pathogenesis of sepsis-induced AKI, including the death of endothelial and epithelial cells, blockage of the renal tubules, changes in the renal microvasculature, and inflammatory processes^15^. The inflammatory reaction is an important pathophysiological feature, and it plays a vital role in sepsis^16^. Renal tubular epithelial cells can be directly affected by inflammatory responses. Systemic inflammation and cytokines in sepsis-induced AKI result in damage to renal tubular epithelial cells through a variety of mechanisms, including immune cell infiltration, microcirculatory disturbance, and renal cell apoptosis.

 Apoptosis is one of the types of cell death and may be triggered by factors such as ischemia, exogenous toxins, and endogenous cytokines^17^ ^,^ ^18^. A growing body of evidence suggests that apoptosis of renal tubular epithelial cells play an important role in sepsis-induced AKI, and apoptosis is one of the major causes of renal damage in sepsis^19^. If apoptotic cells are not rapidly cleared, secondary necrosis occurs in the cells, which produces inflammatory factors and can lead to further organ damage^20^. Therefore, regulation of renal cell apoptosis may be a potential method to effectively treat sepsis-induced AKI^21^.

 MFG-E8 was first isolated from mammalian breast tissue and is a major component of mammalian milk fat globule membranes. Scholars have found that there are two kinds of MFG-E8 mRNA in the rat mammary gland including the 66 KDa long MFG-E8 and the 53KDa short MFG-E8. Short MFG-E8 is widely distributed, while long MFG-E8 is present in activated mouse macrophages, immature dendritic cells, Langerhans cells and keratinocytes. MFG-E8 contains a secretory signal sequence, two N-terminal EGF domains, and two C1 and C2 terminal discoidal domains homologous to clotting factor V and factor VIII. The second EGF domain contains a highly conserved arginine glycine aspartate (RGD) structure that recognizes αvβ3/αvβ5 integrin on macrophages, and the C-terminal discoidal domain is identical to the phosphatidylserine of apoptotic cells. Through this process, MFG-E8 acts as a bridge molecule that promotes macrophages to phagocytose apoptotic cells. Therefore, it plays an indispensable role in eliminating apoptotic mammary epithelial cells^22^. Some diseases, such as Alzheimer, diabetes, sepsis, COPD, and autoimmune diseases, have abnormal MFG-E8 expression^23^ ^-^ ^26^. In the case of sepsis, a large number of immune cells undergo apoptosis and pro-inflammatory cytokine upregulation. Therefore, exogenous MFG-E8 has a protective effect in sepsis.

 NF-κB is a pleiotropic transcription factor that is regulated by many factors^27^. p65 is an essential subunit of NF-κB, and p65 activation levels are often used to represent the extent of NF-κB activation. NF-κB activation is the key to triggering an excessive inflammatory response. Activated NF-κB enters the nucleus and induces the expression of cytokines such as TNF-α, IL-6, and IL-1. The released cytokines can also in turn activate NF-κB, forming a positive feedback loop. In an experimental animal model of septic kidney injury, NF-κB activation is increased. MFG-E8 can promote an anti-inflammatory status indirectly by its extraordinary ability to enhance the phagocytic potentials of apoptotic cells, which in turn ameliorates the inflammation by down-regulating NF-κB. In the present study, NF-κB activation was found in the CLP group, and rmMFG-E8 treatment inhibited NF-κB activation, indicating that inhibiting NF-κB disrupts the production of inflammatory cytokines and mediators. The results of this study demonstrated the activation of NF-κB in sepsis-induced AKI by relative protein expression. Our study found that MFG-E8 plays a protective role in AKI by inhibiting the NF-κB signaling pathway.

 In this study, the possible mechanism of rmMFG-E8 was discussed. Our results show that rmMFG-E8 reduces acute inflammation of the kidneys under sepsis by inhibiting the activation of the NF-κB signaling pathway, reducing inflammation and apoptosis.

## Conclusions

 We have demonstrated that treatment with MFG-E8 having a protective role against sepsis-induced AKI. The underlying mechanism of MFG-E8 on sepsis-induced AKI may be related to inhibiting the release of pro-inflammatory cytokines and mediators, suppressing renal cell apoptosis, and inactivating NF-κB signaling pathway. This evidence suggests that exogenous MFG-E8 may be a potential approach for the treatment of sepsis-induced AKI.
